# Characterizing Expression and Regulation of Gamma-Herpesviral Circular RNAs

**DOI:** 10.3389/fmicb.2021.670542

**Published:** 2021-06-30

**Authors:** Takanobu Tagawa, Daniel Oh, Jerico Santos, Sarah Dremel, Guruswamy Mahesh, Thomas S. Uldrick, Robert Yarchoan, Vishal N. Kopardé, Joseph M. Ziegelbauer

**Affiliations:** ^1^HIV and AIDS Malignancy Branch, National Cancer Institute, Bethesda, MD, United States; ^2^Biological Models Laboratory, Department of Biochemistry and Molecular Biology, College of Medicine, University of the Philippines, Manila, Philippines; ^3^CCR Collaborative Bioinformatics Resource, Center for Cancer Research, National Cancer Institute, National Institutes of Health, Bethesda, MD, United States; ^4^Advanced Biomedical Computational Sciences, Frederick National Laboratory for Cancer Research, Leidos Biomedical Research, Inc., Frederick, MD, United States

**Keywords:** Kaposi sarcoma herpesvirus, Epstein–Barr virus, primary effusion lymphoma, non-coding RNAs, circular RNA, RNA binding protein

## Abstract

Multiple herpesviruses have been recently found to encode viral circular RNAs. Like cellular circular RNAs, these RNAs lack poly-A tails and their 5′ and 3′ ends have been joined, which confers protection from RNA exonucleases. We examined the expression patterns of circular RNAs from Kaposi’s sarcoma herpesvirus (KSHV) in various environments. We performed deep sequencing of circRNA-enriched total RNA from a KSHV-positive patient lymph node for comparison with previous circRNA-Seq results. We found that circvIRF4 is highly expressed in the KSHV-positive patient sample relative to both B cell lines and *de novo* infected primary vascular and lymphatic endothelial cells (LECs). Overall, this patient sample showed a viral circRNA expression pattern more similar to the pattern from B cell lines, but we also discovered new back-spliced junctions and additional viral circular RNAs in this patient sample. We validated some of these back-spliced junctions as circular RNAs with standard assays. Differential expression patterns of circular RNAs in different cell types led us to investigate what cellular factors might be influencing the ratio of viral linear mRNAs to circular RNAs. We found that repression of certain RNA-binding proteins shifted the balance between viral linear mRNAs and circular RNAs. Taken together, examining viral circular RNA expression patterns may become useful tools for discovering their functions, the regulators of their expression, and determining the stage and cell types of infection in humans.

## Introduction

Viruses have been known to express their own non-coding RNAs. Some of these earlier discoveries included HSV’s (Herpes simplex virus) latency-associated transcript (LAT), EBV (Epstein–Barr virus) -encoded small RNAs (EBERs), and KSHV’s (Kaposi’s sarcoma herpesvirus) polyadenylated nuclear (PAN) RNA (Withers et al., 2019; Tagawa et al., 2020), which would now be called lncRNAs (long non-coding RNAs). Studying the functions of these viral lncRNAs was challenging since they do not code for proteins. Then multiple viruses were found to express their own microRNAs (Skalsky and Cullen, 2010). At the beginning stages, expression profiling of cellular and viral miRNAs was the main focus. This led to understanding different expression patterns of miRNAs in diseases like cancer, development stages in *Caenorhabditis elegans*, and cell type specificity (Ambros, 2004; Esquela-Kerscher and Slack, 2006). The viral miRNAs function largely similar to cellular miRNAs and repress target gene expression. Hundreds of target genes of these viral miRNAs have been discovered (Ramalingam et al., 2012; Vojtechova and Tachezy, 2018; Židovec Lepej et al., 2020). Some of these viral miRNAs target cellular and viral transcripts and serve to regulate apoptosis, cytokine responses, and immunogenicity (Skalsky and Cullen, 2010; Tagawa et al., 2020). The relevance, functions, and mechanisms of non-coding RNAs in virology has been realized in recent decades. A recent addition to this field of non-coding RNAs is circular RNAs (circRNAs).

Covalently closed circular RNAs were first discovered in plant viroids (Sanger et al., 1976). CircRNAs lack free 5′ and 3′ ends, which allow them to be protected from exonucleases. Since these circRNAs lack poly-A tails, they were typically discarded during poly-A RNA enrichment protocols that are used before RNA sequencing (Kristensen et al., 2019). Use of ribosomal RNA-depleted total RNA sequencing rather than poly-A RNA sequencing has improved the sensitivity to detect circRNAs. Other methods to further enrich for circRNAs include treating RNA samples with RNase R, an exonuclease, to degrade linear RNAs but preserve circRNAs, and RPAD [RNase R treatment, polyadenylation, and poly(A) + RNA depletion], which further purifies RNA samples for circular RNAs (Pandey et al., 2019). New bioinformatics tools have allowed researchers to look for these back-spliced junctions, a unique marker of circRNAs (Hansen, 2018). Much like the beginning of the miRNA field, initial studies have focused on the expression profiling of cellular and viral circular RNAs in different diseases, cell types, and infection (Tagawa et al., 2018; Toptan et al., 2018; Ungerleider et al., 2018; Huang et al., 2019; Zhao et al., 2019; Abere et al., 2020b).

Early work on cellular and viral circRNAs in the context of infection have found differential expression patterns and some limited examples of functions, including a cellular circRNA that inhibits infection and viral circRNAs that alter cell growth (Tagawa et al., 2018; Toptan et al., 2018; Huang et al., 2019; Liu et al., 2019; Ungerleider et al., 2019, 2018; Zhao et al., 2019; Abere et al., 2020a, b). Here we focus on circRNAs in the context of Kaposi sarcoma herpesvirus (KSHV) and Epstein–Barr virus (EBV). Through deep sequencing of a biopsy from a patient with KSHV infection and other types of KSHV infection, such as reactivation of KSHV-positive cell lines and *de novo* infection, we discovered new viral circRNAs. We also found complexity in the splicing events leading to the synthesis of these viral circRNAs. We examined the biogenesis and subcellular localization of these circRNAs. These viral circRNAs represent potential biomarkers for disease and therapeutic targets in viral diseases.

## Materials and Methods

### Cell Culture

Human dermal lymphatic endothelial cells (LECs) were obtained from PromoCell and incubated in EGM-2 medium (Lonza) for up to five passages. LECs were fed every 2 days with fresh media. Cells were used for experiments between three and five passages. SLK cell lines were maintained in DMEM medium supplemented with 10% FBS (Gibco), penicillin (100 U/ml; Gibco), and streptomycin (100 mg/ml; Gibco). SLK cell lines that express circRNAs constitutively (Tagawa et al., 2018) were kept under selection with G418 (250 μg/ml; Gibco). The KSHV-infected iSLK cell line (BAC16 strain) is a gift from Rolf Renne and is maintained in DMEM supplemented with 10% FBS, penicillin, streptomycin, hygromycin (1,200 μg/ml; Gibco), puromycin (1 μg/ml; Gibco), and G418 (250 μg/ml). TREx-BCBL1-RTA cell line (gift from Jae Jung) is maintained in RPMI medium supplemented with 10% FBS, penicillin, streptomycin, and 2-mercaptoethanol (55 μM; Gibco). Cells were cultivated at 37°C in a 5% CO_2_ incubator.

### KSHV Infection and Reactivation

BAC16 virus was prepared with iSLK cells induced for lytic cycle with doxycycline (1 μg/ml; Thermo Fischer Scientific) and sodium butyrate (1 mM; MilliporeSigma) for 3 days. Collected supernatants were cleared of debris with centrifugation at 500 × *g* for 5 min, filtered (Rapid-Flow 0.45 μm filter; Thermo Fischer Scientific), and concentrated with ultracentrifugation (2.5 h at 4°C at 50,000 × *g* with SW 32 Ti rotor; Beckman Coulter). *De novo* infections of LECs were carried out by diluting concentrated supernatant in EGM-2 medium at a multiplicity of infection (MOI) of 15, 30, or 45, as determined by LANA copy number, unless otherwise mentioned. MOI values of 0, 15, 30, and 45 correspond to 0, 25, 53, and 70% of cells being infected based on GFP, a surrogate marker encoded in BAC16. Polybrene (Millipore Sigma) was added at 8 μg/ml for infections. Virus supernatant was washed off after overnight incubation and replaced with fresh EGM-2 media. TREx-BCBL1 RTA cells were induced for the lytic cycle with doxycycline (1 μg/mL; Thermo Fisher Scientific) for 2 days. iSLK-BAC16 cells were induced for lytic cycle with doxycycline (1 μg/mL) and sodium butyrate (1 mM) for 2 days.

### RNA Quantitation by RT-PCR

Total RNAs were extracted with Direct-Zol kit (Zymo Research). For RNase R resistance assay, 3 μg of total RNA were treated with 20 U of RNase R (Lucigen) and 20 U of RiboLock RNase (Thermo Fisher Scientific) for 30 min at 37°C, followed by purification with RNA Clean and Concentrator 5 kit (Zymo Research). For certain experiments, total RNAs were separated into nuclear and cytoplasmic fractions according to manufacturer’s guidance in Cytoplasmic and Nuclear RNA Purification Kit (Norgen Biotek). RNAs were reverse transcribed with ReverTra Ace (TOYOBO). Quantitative PCR was performed with Thunderbird SYBR qPCR kit (TOYOBO) on a StepOnePlus real-time PCR system (Thermo Fischer Scientific). Relative transcript levels were computed using the threshold cycle (ΔΔCt) method. Absolute quantification was performed with droplet digital PCR on QX200 Droplet Digital PCR (ddPCR) System (Bio-Rad). Primers are listed in Supplementary Table 1.

### circRNA-Seq of a Clinical Sample

A lymph node biopsy was obtained from a patient of primary effusion lymphoma (PEL) with a confirmed history of HIV and KSHV co-infection (PEL5-LN). Pathological analysis determined that the same lymph node also contained PEL at time of biopsy. Plasma HIV-1 mRNA was measured by real-time quantitative RNA polymerase chain reaction using Amplicor HIV-1 Monitoring Kits (Roche Diagnostic Systems). KSHV tumor status was confirmed by staining for latency-associated nuclear antigen (LANA) (anti-ORF73 rat mAb, Advanced Biotechnologies). The patient was enrolled in an Institutional Review Board approved protocol at the National Cancer Institute (NCTNCT00006518). The patient gave written informed consent.

For circRNA-Seq, total RNA was purified with miRNeasy Kit (Qiagen) and enriched for circular RNA with RNase R (Lucigen) according to manufacturer’s instructions. The library was generated using TruSeq Stranded Total RNA System (Illumina). Paired-end 150 bp RNA-Seq (NextSeq, illumina) sequencing reads were aligned and mapped with STAR (Dobin et al., 2012) to the complete genome sequence of KSHV, NC_009333 (KSHV), and NC_007605 (EBV) with two-pass alignment for accurate mapping to novel splicing junctions. The chimeric alignments from STAR were subjected to CIRCexplorer2 (Zhang et al., 2016) to detect circRNAs (Supplementary Tables 2, 3). The sequencing data was deposited to GEO (Gene Expression Omnibus; GSE165529). For comparison, publicly available data sets were analyzed simultaneously: GSE124711 (reactivated BCBL1) (Ungerleider et al., 2019), GSE117798 (reactivated BC1) (Toptan et al., 2018), and PRJEB31253 (European Nucleotide Archive, rKSHV.219-infected LEC) (Gramolelli et al., 2020).

**FIGURE 1:**
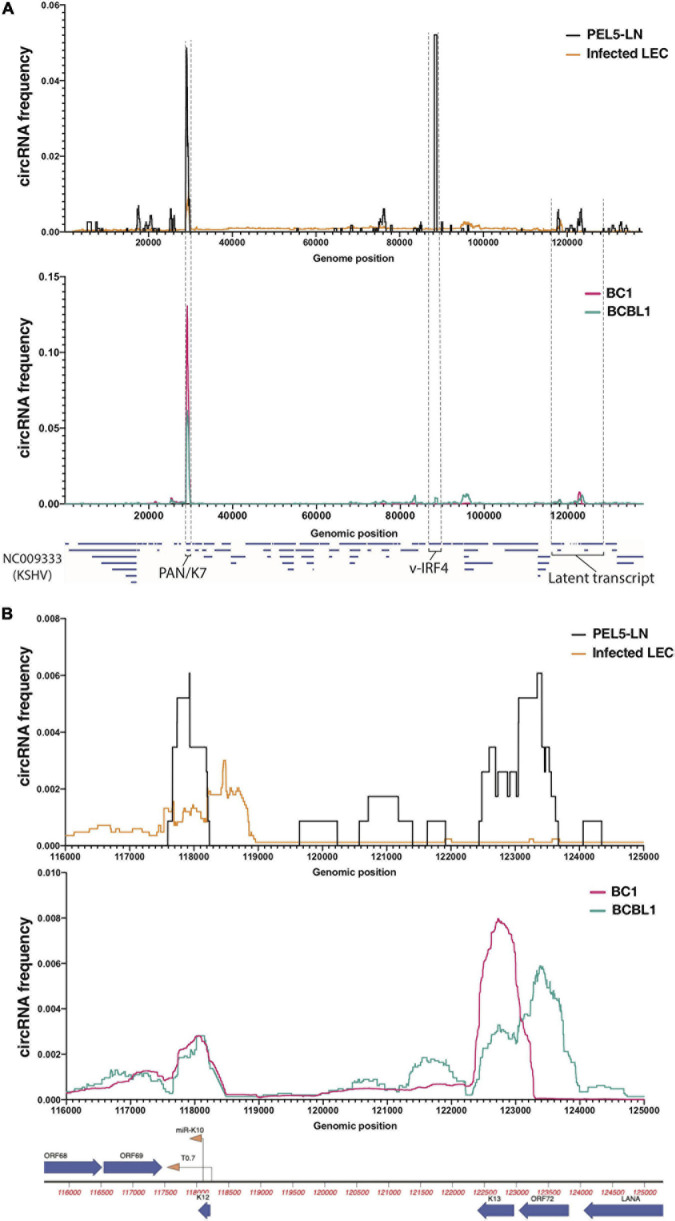
Profiles of KSHV circular RNAs detected by RNA-Seq. Frequency histograms of KSHV circular RNAs in a lymph node of KSHV-positive patient (PEL5-LN) compared with *de novo* infected LECs (above), and reactivated KSHV-positive B cell lines (below). Detected viral circular RNA frequencies based on back-splice junctions (BSJ) reads (see section “Materials and Methods” for detail) from circRNA-Seq (PEL5-LN and B cell lines) or Ribosomal RNA-depleted RNA-Seq (LECs) are shown as percentages of total detected viral BSJ reads (y-axis) across the whole KSHV genome **(A)** or the KSHV latent transcript region **(B)** (x-axis).

CIRCExplorer2 outputs BED (Browser Extensible Data) files describing 5′ and 3′ ends of BSJs as well as read counts for each BSJ (Supplementary Table 3). To comprehend global viral circRNA expression profiles, we calculated circRNA frequency through the KSHV/EBV genome. Unlike mRNA-originated reads, BSJ-containing reads only accounts for flanking region of circRNAs of interest. To compensate for this problem, we defined sequences between 5′ and 3′ ends of each detected BSJ as circRNA-coding region and calculated coverage at single nucleotide resolution as in the Coverage Plot of Integrative Genomics Viewer (IGV) (Robinson et al., 2017) and normalized to area under curve for comparison of all samples and shown as percentages (Figure 1).

### Deep-Sequencing of Divergent RT-PCR Amplicons

TREx-BCBL1 RTA cells and iSLK-BAC16 were reactivated for the lytic cycle for 3 days and total RNA was extracted, treated with RNase R, and reverse-transcribed as previously described. cDNA was amplified with KOD One polymerase (Toyobo) and libraries were prepared with NEBNext Ultra II DNA library Prep kit (New England Biolabs). 300 nt single-end reads (MiSeq, illumina) were mapped to NC_009333 and circRNAs were detected by CIRCexplorer2 (Supplementary Tables 2, 4) and normalized as in “circRNA-Seq of a clinical sample.” The sequencing data was submitted to GEO (GSE165529).

### Exon-Wise Quantitation of Transcript Expression Levels

We reanalyzed our previous transcriptome analysis of KSHV-infected HUVECs (GSE120045) using Partek Genomics Suite’s mRNA quantification function (Partek). Exon-wise read counts are normalized to RPKM (reads per kilobase in million reads) and fold change was calculated based on RPKMs. Locations of known human circRNAs were imported from circBase^1^.

### Transcriptome Analysis of SLK Cell Lines That Ectopically Express Viral circRNAs

Stable SLK cell lines expressing the circGFP, circORF34 (kcirc54), or circORF35-36 (kcirc55) (Tagawa et al., 2018) were harvested for total RNA with Direct-Zol (Zymo Research) and library was generated using TruSeq Stranded Total RNA System (Illumina, *n* = 4). Paired-end 150 nt RNA-Seq (HiSeq, Illumina) sequencing reads were aligned and mapped with STAR to GRCh38 and NC_009333. DEG (differentially expressed gene) analysis was performed with DESeq2 (Love et al., 2014; Supplementary Table 5). Ingenuity Pathway Analysis (IPA, Qiagen) was performed for DEGs (*p*-value < 0.05) and Regulator Effects are shown in Figure 4C. The sequencing data was deposited to GEO (GSE165529).

**FIGURE 2:**
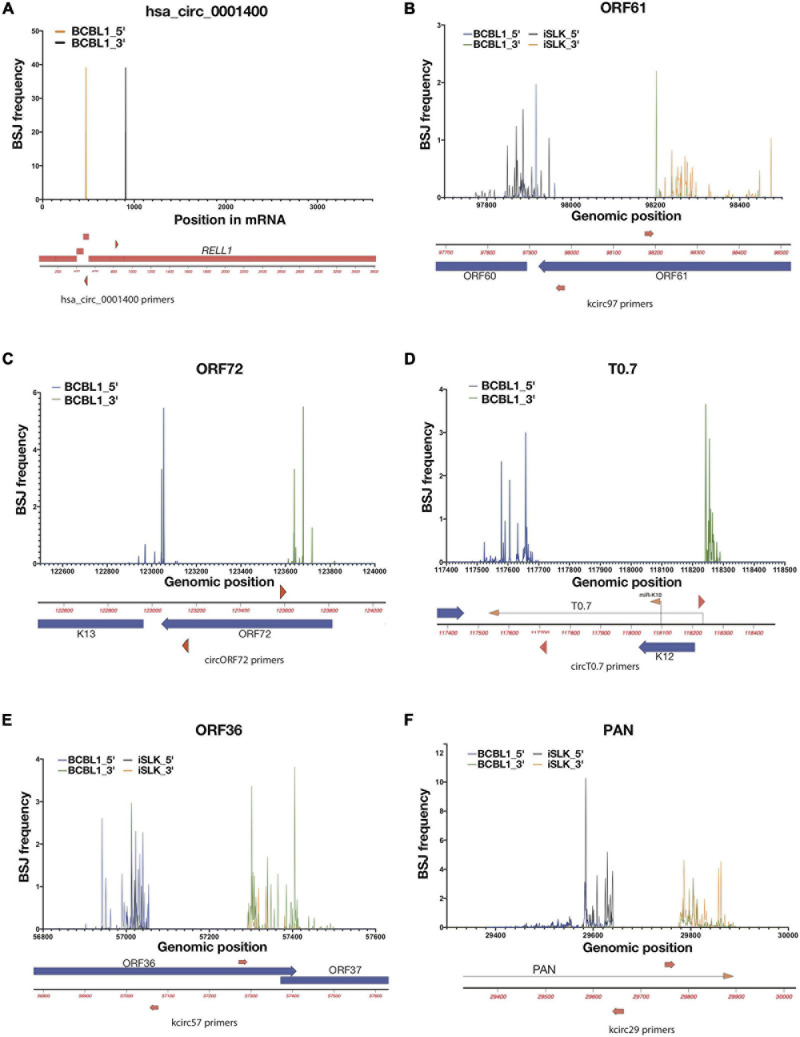
Variabilities of back-splice junction (BSJ) loci in KSHV. Frequencies of 5′ and 3′ positions of back-spliced junctions detected in reactivated iSLK-BAC16 (iSLK) and TREx-BCBL1 RTA (BCBL1) cell lines. Total RNAs from KSHV-positive cell lines were RNase R-treated and RT-PCR was performed to amplify BSJ regions. Amplicons were sequenced and mapped to KSHV genome (NC_009333), and 5′ and 3′ BSJs are shown as percentages total detected viral BSJ reads (y-axis) for each divergent primer pairs (shown as red triangles or arrows, Supplementary Table 1) across flanking regions of circRNA-specific primers (x-axis). Human circRNA hsa_circ_0001400 serves as positive control of this analysis **(A)**. For viral circRNAs, genomic regions around ORF61 **(B)**, ORF72 **(C)**, T0.7 **(D)**, ORF36 **(E)**, and PAN **(F)** are shown with primer locations.

**FIGURE 3:**
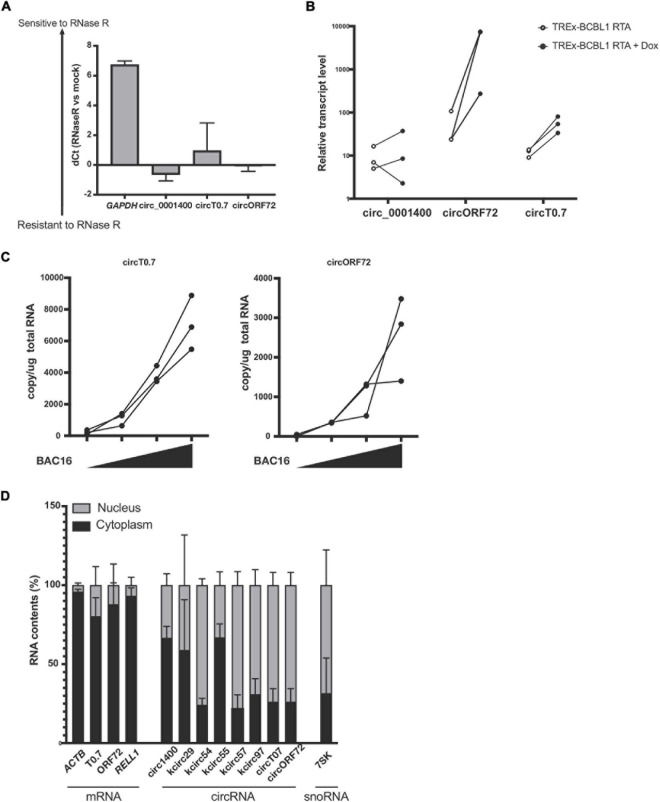
Expression of circRNAs in KSHV T0.7 and ORF72 ORFs. **(A)** circRNA resistance to RNase R-mediated degradation. Total RNA from reactivated TREx-BCBL1 RTA cells was treated with RNase R and subjected to RT-qPCR. Delta of Ct values between mock and RNase R-treated samples are shown (RNase R degradation is denoted by higher dCt values). *GAPDH* and hsa_circ_0001400 are controls of linear and circular RNA, respectively. *n* = 3. **(B)** Expression of kshv circT0.7 and circORF72 in reactivated TREx-BCBL1 RTA cells for 2 days. Extracted total RNAs were subjected to RT-qPCR with divergent primers. *GAPDH* was used as internal control (*n* = 3). **(C)** Expression of circT0.7 and circORF72 in *de novo* infected LECs with increasing amounts of KSHV BAC16 at MOI of 0, 15, 30, or 45, as determined by LANA copy number. Total RNAs were extracted at 3 days post infection and subjected to RT-ddPCR with divergent primers. Absolute copy numbers were quantitated and normalized to 1 μg total RNA for each condition (*n* = 3). **(D)** Localization of viral circRNA in reactivated TREx-BCBL1 RTA cells. Nuclear and cytoplasmic RNA contents were purified after reactivation and subject to RT-qPCR. Delta Ct values between nuclear and cytoplasmic amounts of each RNA species were calculated and are shown as percentages (*n* = 3). Error bars indicate standard deviation.

**FIGURE 4:**
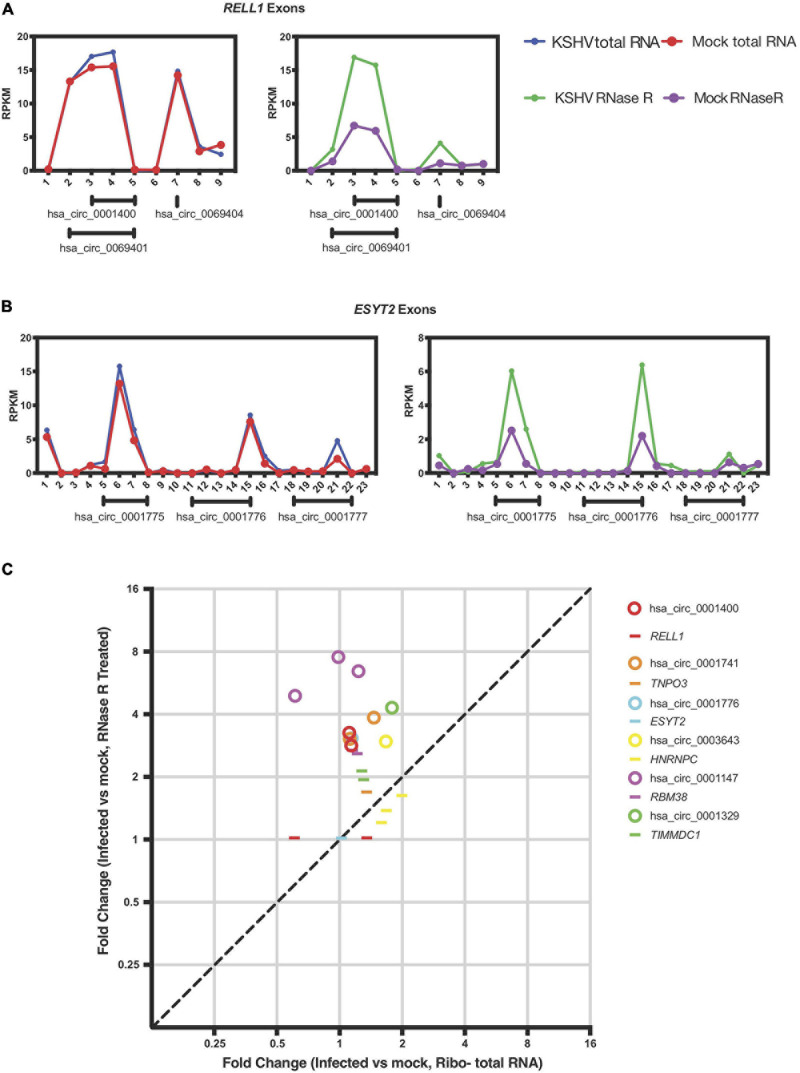
KSHV induces circRNA expression post-transcriptionally. **(A,B)** Exon-wise analysis of read-counts upon KSHV infection in *RELL1* **(A)** and *ESYT2* **(B)** loci. Shown are RPKM (reads per kilobase in million reads) of total RNA Seq (left) and circRNA-Seq (right). Numbers in x-axis corresponds to exon number from 5′ ends. Under each panel, locations of human circRNA in circBase are shown. **(C)** Exon-wise fold-change of select genes comparing infected and non-infected human primary endothelial cells. Circles are exons that are part of circRNAs and lines are exons that are not known to be circRNAs according to circbase. Only exons with aligned read counts > 0.05 RPKM are shown.

### Knock-Down of RNA-Binding Protein-Coding Transcripts With siRNAs

To select candidates of knock-down, we merged screening datasets from Li et al. (2017) for regulators of circRNA production, high-throughput RNA-binding protein identifications (Queiroz et al., 2019; Urdaneta et al., 2019), and transcriptome analysis of *de novo* KSHV-infected human umbilical vascular endothelial cells (HUVECs) (Tagawa et al., 2018; Supplementary Table 6). SLK, iSLK-BAC16, and LECs were transfected with 20 nM of ON TARGETplus SMARTpool siRNAs (Horizon) with RNAiMAX (Thermo Fisher Scientific, SLK and iSLK-BAC16) or DharmaFECT 1 (Horizon) in Opti-Mem I (Gibco) according to manufacturer’s guidance and incubated for 24 h. SLK cells were infected with BAC16 KSHV at an MOI of 0, 1, or 2, with polybrene (8 μg/ml). At 24 h post-infection, the virus containing media was replaced. 48 h after replacing the media, total RNA was extracted using Direct-zol (Zymo Research).

## Results

### Viral circRNA Expression Profiles in Infected Cells *in vitro* and *in vivo*

Relevant cell types for KSHV infection include B cells and endothelial cells, and infections can lead to pathology of endothelial origin (Kaposi’s sarcoma) or B cell origin (PEL and multicentric Castleman disease) (Yarchoan and Uldrick, 2018). Through circRNA-Seq, we and others have screened for KSHV-encoded circRNAs in latently infected endothelial cells as well as B cell lines reactivated for lytic cycle *in vitro* (Tagawa et al., 2018; Toptan et al., 2018). Comprehensive understanding of viral circRNAs expression *in vivo* in clinical settings, however, is still incomplete. We examined expression patterns of viral circRNAs in a KSHV-positive patient and compared with patient-derived cell lines and cell culture infections in primary cells. We extracted total RNA from a lymph node biopsy of a KSHV-positive PEL patient (“PEL5-LN”) and enriched for circRNAs with RNase R treatment and ribosomal RNA depletion (Figure 1). The lymph node used was determined to be KSHV-positive by LANA immunohistochemistry. Previous analysis of this patient sample found multiple KSHV lytic genes expressed at high levels [patient 5 in Tagawa et al. (2018)]. We also analyzed publicly available RNA-Seq data of reactivated KSHV-positive PEL cells [circRNA-Seq of BC-1 (Toptan et al., 2018) and BCBL-1 (Ungerleider et al., 2019)] as well as KSHV-infected primary LECs (Ribo^–^ RNA-Seq) (Gramolelli et al., 2020; Figure 1). LECs are known to spontaneously enter lytic infection (most KSHV circRNAs are expressed in the lytic cycle) upon *de novo* KSHV infection (Gramolelli et al., 2020). We employed CIRCexplorer2 (Zhang et al., 2016) to detect circRNAs. The algorithm takes short-read sequencing data such as illumina and detects sequencing reads that contain back-splice junctions (BSJs) (Supplementary Figure 1A). We mapped aforementioned Ribo- and circRNA-Seq data to the KSHV genome (NC_009333) and subjected mapped reads to the CIRCexplorer2 program [see section “Materials and Methods” and Tagawa et al. (2021) for detail]. Figure 1A shows the histograms of detected circRNAs throughout KSHV genome. We found many BSJs in the PAN region in LECs and in the lymph node RNA from this patient with KSHV disease [Figure 1A, circK7/PAN (Tagawa et al., 2018; Toptan et al., 2018)]. It should be noted that the expression of the KSHV transcript called polyadenylated and nuclear transcript, PAN, is the most abundant transcript in many KSHV lytically infected cells (around 500,000 copies per cell, 80% of all poly-A RNAs) (Sun et al., 1996). However, a striking difference in these two contexts of infection was observed when finding the BSJs from circ-vIRF4. In the patient’s RNA sample, we found robust expression of circ-vIRF4, but this was much less than in PEL cell lines (BC-1, BCBL-1) and in the endothelial cell infection in cultures under either at latent (Tagawa et al., 2018) or lytic conditions (Figure 1A and Supplementary Table 3).

The latency locus in KSHV contains many transcripts, including polycistronic transcripts, protein-encoding transcripts, miRNAs, and other non-coding transcripts (Ajiro and Zheng, 2014). In addition to previously reported circRNAs at the PAN and vIRF4 loci (Tagawa et al., 2018; Toptan et al., 2018; Ungerleider et al., 2019), in the PEL cells, we found two regions that contain BSJs (T0.7 and K13/ORF72) (Figure 1B). We also found evidence of KSHV circRNAs at this locus in the LECs, B cell lines, and lymph node sample, but their expression appears to vary. The circRNAs at T0.7 (viral long non-coding RNA) region is consistently expressed among all samples whereas ORF72 circRNAs appears only in PEL5-LN and BCBL1. Overall, PEL5-LN showed the pattern in parallel to BCBL1 rather than BC1, even though both cell lines are derived from PEL patients (Figure 1B).

Co-infection of B cells with KSHV and EBV is common is individuals with PEL (Yarchoan and Uldrick, 2018). PEL5-LN was also co-infected, and we identified an isoform of the reported EBV circRNA (Ungerleider et al., 2018), ebv-circRPMS1_E4_E2 (Supplementary Table 3). Taken together, these results show a variety of expression patterns of circRNAs in a clinical sample and patient-derived cell lines. Some of these expression patterns might be useful as biomarkers for infection and disease and take advantage of the fact that these circRNAs are more stable than linear RNAs.

### Variable Ends of Viral circRNAs

The frequency histograms in Figure 1 represent the location of back-spliced junctions relative to the viral genome but fail to reveal the complexities of these viral back-spliced areas. We have closely examined the variability in back-spliced junctions by investigating RNA sequencing reads. In addition, we have amplified back-spliced junctions with divergent PCR primers that selectively amplify circRNAs (Supplementary Table 1) and then cloned and Sanger sequenced these PCR products (Tagawa et al., 2018). Different ends of BSJs were observed for certain KSHV circRNAs (Tagawa et al., 2018; Supplementary Figure 1A), whereas EBV circRNA circBHLF1 shows more variability upon lytic induction when linear and circular BHLF1 is highly expressed (Ungerleider et al., 2019; Supplementary Figure 1B and Supplementary Table 3). We further examined PCR products using deep sequencing to quantitatively determine if dominant BSJ positions exist, using CIRCexplorer2 for circRNA detection. We focused on two samples: 1. Lytically induced BCBL1 cells, which are patient-derived KSHV-positive cells 2. Lytically-induced iSLK-BAC16 cells, which are infected with a recombinant form of KSHV. We PCR- amplified specific BSJs using divergent primers. These PCR products were then used for deep sequencing. In contrast to a cellular circRNA (hsa_circ_0001400, Figure 2A), we have found a range of variability in these viral BSJs. Figure 2 shows that, for KSHV circRNAs at ORF61 (circORF61), we found several major positions for the 5′ and 3′ ends of BSJs (Figure 2B). A similar pattern of variability was observed for the KSHV circRNA around the ORF72 transcript with 2–3 major peaks (Figure 2C). Other viral circRNAs at T0.7, ORF36 (circORF36), and PAN (circPAN) showed many different positions for the 5′ and 3′ ends of the BSJs (Figures 2D–F). For circPAN, multiple major peaks appeared in both BCBL1 and iSLK cells, though, for other circRNAs, different splice junction positions seemed to be favored more in one cell type, compared to the other cell type. This BSJ variability suggests that the exact junction is not important for viral infection, but rather the other regions of the circRNAs outside of the junction are crucial.

### KSHV circRNAs in the Latent Transcript Coding Region

We validated potential circRNAs based on BSJ-containing reads identified in total RNA-seq with additional experiments. A common method is to use divergent primers that amplify circRNAs, but do not produce PCR products from linear transcripts. An additional component involves treating RNA samples with RNase R and measuring protection from RNase R. As shown in Figure 3A, we found protected PCR products with divergent primers for circRNAs: human hsa_circ_0001400 (also known as circRELL1), KSHV circT0.7, and KSHV circORF72. The results demonstrated that these circRNAs were protected from RNase R degradation, but a control linear RNA, *GAPDH*, was readily degraded by RNase R. Similarly, we validated the expression of circRNAs and found that novel KSHV circT0.7 and KSHV circORF72 expression increased by 10- to 100-fold in lytically-induced KSHV cells, while the human circ_0001400 was unchanged between the latent and lytic cycles in the inducible BCBL1 cell line TREx-BCBL1 RTA (Figure 3B). Similarly, both viral circRNAs were detected in *de novo* infected LECs in a virus dose-dependent manner (Figure 3C).

### Subcellular Localization of KSHV circRNAs

In order to begin thinking about potential functions of circRNAs, we examined the subcellular localization of human and viral circRNAs (Figure 3D), as certain functions are restricted to certain areas. It has been reported that certain viral circRNAs are located both in nucleus and cytoplasm, unlike the typical human exonic circRNAs (Toptan et al., 2018). After fractionating cells into nuclear and cytoplasmic components, we measured circRNAs using divergent PCR primers. In general, we found most human circRNAs to be predominantly in the cytoplasmic fraction. However, KSHV circRNAs displayed multiple patterns: 1. mostly cytoplasmic 2. mostly nuclear 3. almost equal distribution between these two areas. KSHV circT0.7, KSHV circORF72, and linear mRNAs *ACTB* and *RELL1* were largely cytoplasmic. Human circ_0001400 and a couple of KSHV circRNAs were mostly cytoplasmic and the remaining KSHV circRNAs were mostly nuclear. Subcellular localization can be a useful step in formulating hypotheses about the potential function of circRNAs since RNA-RNA or RNA-protein interactions may be restricted to one of the two locations. Additionally, certain biological processes and pathways are also restricted to a specific subcellular compartment.

### Regulation of Viral circRNA Expression by Human RNA-Binding Proteins

Previous reports in the literature and screens for factors that may regulate circRNA biogenesis were integrated with our previous expression data of cellular genes that change with viral infection (Kristensen et al., 2019). We hypothesized that viral infection may alter expression or activity of cellular factors that would promote or inhibit generation of circRNAs. We first re-examined the previously reported dataset with latently infected primary endothelial cells (HUVECs) for RNA expression differences with KSHV infection at the mRNA exon level. To test whether KSHV infection alters splicing patterns of circRNAs, we selected human circRNAs that were upregulated upon KSHV infection and analyzed the back-splicing and normal splicing patterns in these regions (Tagawa et al., 2018). The *RELL1* gene locus contains multiple circRNAs, including circ_0001400, one of the most highly upregulated human circRNA upon infection. As shown in Figure 4A, KSHV infection correlated with increased read counts on exons containing circRNAs based on circBase sequence information (see text footnote 1), only in RNase R treated sequencing. The same was observed in the *ESYT2* transcript (Figure 4B) and other transcripts (Figure 4C). When each exon is treated as a transcript, expression level fold changes of exons that are not known to encode circRNAs upon KSHV infection are comparable with or without RNase R treatment (Figure 4C, depicted as lines). In contrast, circRNA-encoding exons’ fold changes are larger in RNase R-treated sequencing compared to total RNA-Seq (Figure 4C, depicted as circles), suggesting that KSHV infection can promote back-splicing events to increase the circRNA levels for certain genes.

**FIGURE 5:**
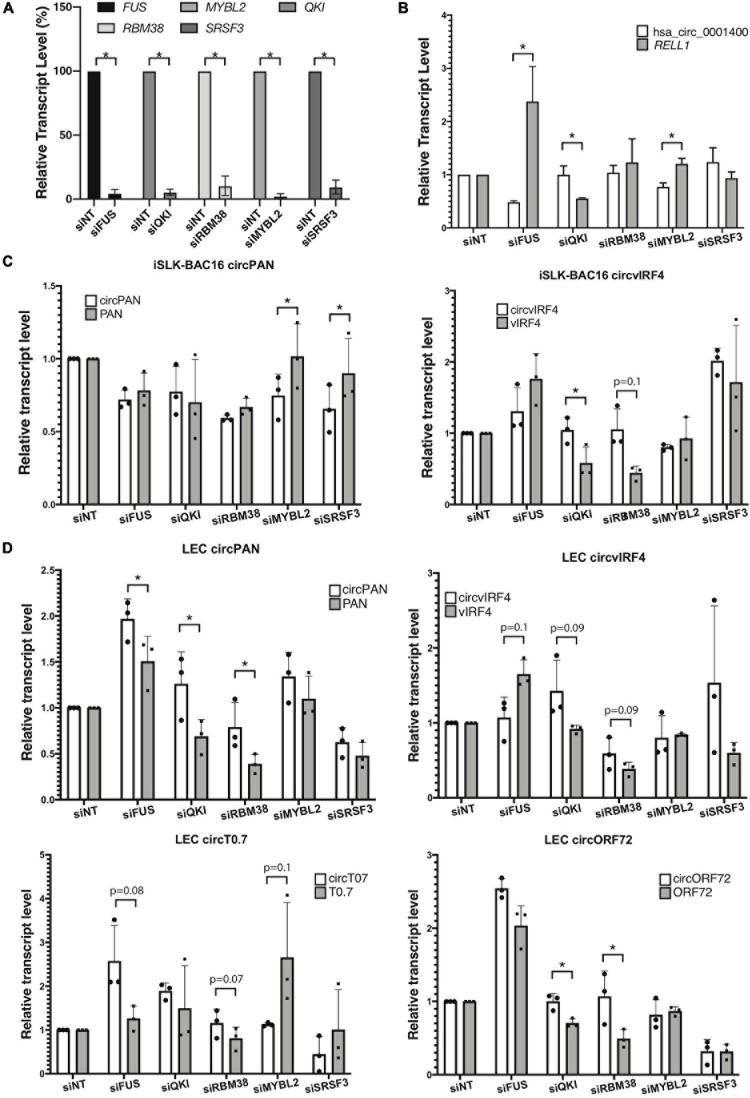
RNA-binding proteins and production of viral circRNAs. RNA-binding proteins (RBPs) were knocked-down with siRNA pools and circular RNAs and their linear mRNAs counterparts were quantitated. **(A,B)** KSHV-negative SLK cells were knocked-down for RBPs and RBP-coding transcript levels (*n* = 4) **(A)**, and hsa_circ_0001400 and *RELL1* expression levels (*n* = 3) **(B)** were quantitated with RT-qPCR. Expression levels were normalized to non-targeting siRNA (siNT) control. 18 S RNA was used as internal control. **(C)** iSLK-BAC16 cells were knocked-down for RBPs, reactivated for lytic cycle, and viral circRNAs and their linear counterparts were quantitated with RT-ddPCR. Absolute copy numbers were quantitated and normalized to non-targeting siRNA control. **(D)** Primary LECs were knocked-down for RBPs, *de novo* infected for 3 days at MOI of 30, and viral circRNAs and their linear counterparts were quantitated with RT-ddPCR. Absolute copy numbers were quantitated and normalized to siNT control (*n* = 3). Error bars indicate standard deviation. Statistical significances were calculated by paired ratio *t*-test. *p < 0.05.

To understand the functional significance of certain RNA-associated proteins in the regulation of circRNA biogenesis, we inhibited expression of specific cellular factors and measured changes in the ratio of linear RNA to circRNA levels, in the context of KSHV infection. To identify candidate RBPs that would be studied first, we selected genes coding for proteins that have been reported to bind to RNA, regulate back-splicing, and be differentially regulated upon *de novo* infection (Li et al., 2017; Tagawa et al., 2018; Kristensen et al., 2019; Queiroz et al., 2019; Urdaneta et al., 2019; Supplementary Table 6). These are namely *FUS*, *QKI*, *MYBL2*, and *RBM38*, as well as a splicing factor *SRSF3*, known to interact with a KSHV key lytic gene product, called ORF57/MTA (Majerciak et al., 2014). As a proof of concept, we first performed the knock-down experiments in an SLK epithelial cell line (Figure 5A). Repression of human *FUS* decreased the amount of human circ_0001400 and increased the amount of the linear *RELL1* mRNA (Figure 5B). Repression of *MYBL2* showed a similar pattern. We observed an opposite pattern when QKI was repressed, with an increase in circ_0001400 levels and a decrease in the linear transcript. Next, to evaluate effect of these RBPs on viral circRNA expression levels, we performed RNAi-mediated knock-down in iSLK-BAC16 cells during lytic reactivation as well as in *de novo* infected LECs (Figures 5C,D). In iSLK-BAC16 cells, transfection of siMYBL2 and siSRSF3 decreased circPAN levels compared to linear PAN whereas the same siRNAs had no significant effect for circvIRF4, suggesting that MYBL2 and SRSF3 positively regulate production of circPAN, but not circvIRF4. In contrast, QKI and RBM38 appear to repress circvIRF4 levels without shifting the ratio between linear and circular PAN (Figure 5C). These specificities were observed in infected LECs (Figure 5D). Changing levels of MYBL2 shifted the balance toward circRNAs, but only for circT0.7. FUS positively regulated circvIRF4, as with hsa_circ_0001400 (Figures 5B–D), and negatively regulated circRNA at PAN, T0.7, and ORF72 (Figure 5D). QKI and RBM38 consistently behaved as negative regulators of most of viral circRNAs. Taken together, we identified RBPs that shift balance between linear and circular viral transcripts generally (QKI, RBM38) and specifically (FUS, MYBL2, SRSF3). These investigations demonstrate that the biogenesis of specific circRNAs is regulated by different RBPs. This adds another level of gene expression regulation at the post-transcriptional step.

### Host Gene Changes Induced by KSHV circRNAs

**FIGURE 6:**
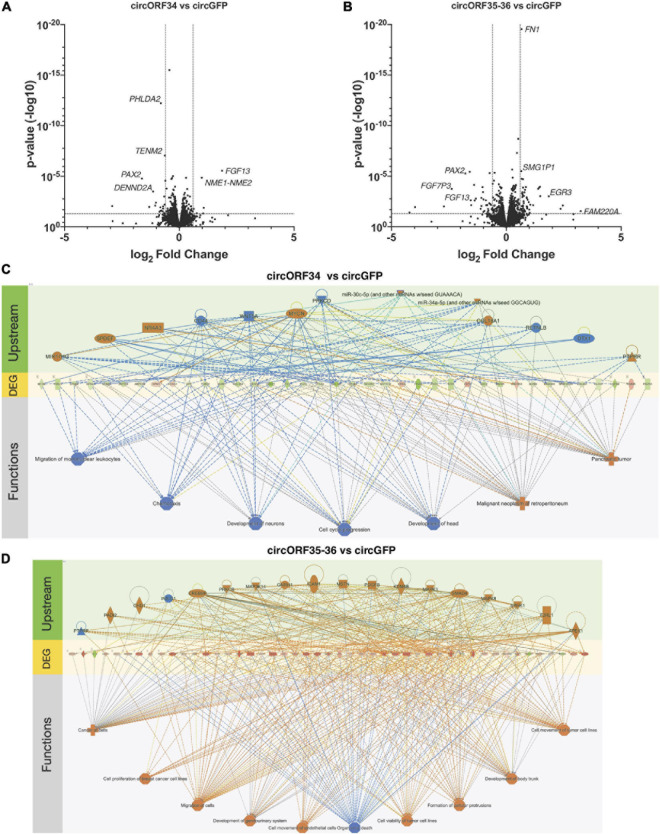
Transcriptomes change by specific viral circRNAs. KSHV-negative SLK cell lines that constitutively express select KSHV circRNAs were subjected to total RNA-Seq analysis. **(A,B)** Volcano plots showing differential gene expressions in circORF34- **(A)** or circORF35-36- **(B)** expressing cell lines compared to circGFP (negative control). Adjusted *p*-value of 0.05 and fold-change of | 1.5| are marked. **(C,D)** Regulator effects analysis in Ingenuity Pathway Analysis package are shown. Upstream regulators and downstream functions are predicted based on the genes differentially expressed by circORF34 **(C)** or circORF35-36 **(D)** (*n* = 3). Blue colors denote repression and orange colors denote stimulation. Green and red shades denote the level of increased or decreased expression induced by the circORF34 or circORF35-36. Dotted lines denote indirect regulations and solid lines show known direct regulations.

Loss of function experiments is difficult since most approaches are dependent on targeting the sequence of the BSJs. This is challenging with many viral circRNAs as their BSJs are variable and it is not possible to target a BSJ of interest with a single siRNA or antisense oligonucleotide. As an alternative approach, we ectopically expressed certain KSHV circRNAs and measured changes to human gene expression using RNA sequencing. Specifically, we constitutively expressed circORF34 and circORF35-36 [termed kcirc54 and kcirc55, respectively, in Tagawa et al. (2018)] at physiological levels in SLK cells (Tagawa et al., 2018) and identified differentially expressed genes by using cells expressing circGFP (Kramer et al., 2015) as a control. We detected reads that mapped to ORF34, 35, and 36, confirming the expression of viral circRNAs (Supplementary Table 5). Constitutive ectopic expression of both circORF34 and circORF35-36 reduced the expression of *PAX2*, compared to the circGFP control cell line (Figure 6A). Additionally, circORF35-36 increased the expression of *Early Growth Response 3* mRNA (induced during mitosis) and circORF34 increased *Fibroblast growth factor 13* mRNA. We broadly investigated the gene expression changes induced with these individual KSHV circRNAs using a pathway analysis tool. Figures 6C,D displays the differentially expressed genes in the center with predicted upstream regulators of these genes displayed above, and the downstream phenotypic consequences presented below. We note that in Figure 6C the cell cycle progression pathway is predicted to be repressed overall based on the differentially expressed genes. In contrast, Figure 6D indicates a prediction of increased cell proliferation. These predictions are consistent with our previous cell growth assays that showed decreased cell proliferation with ectopic expression of KSHV circORF34 and increased proliferation with ectopic expression of KSHV circORF35-36 (Tagawa et al., 2018).

## Discussion

Viral non-coding RNAs have a wide range of functions and are often crucial for successful infection. Novel circRNAs were recently identified in various DNA viruses including KSHV, EBV, HPV (human papillomavirus), and MCPyV (Merkel cell polyomavirus) (Tagawa et al., 2018; Toptan et al., 2018; Ungerleider et al., 2018; Huang et al., 2019; Zhao et al., 2019; Abere et al., 2020b). Circular RNAs have gene regulatory functions in human cells and viral circRNAs can be additional regulators of host-virus interactions (Kristensen et al., 2019). Functional analysis of viral circRNAs is still quite limited due to unconventional splicing donor/acceptor sites that are flanking BSJs and the high variability of BSJs. These characteristics limit our ability to comprehensively catalog viral circRNAs and perform gain- or loss-of-function studies of circRNAs. Here, we performed comparative analysis of viral circRNAs across multiple cell lines and a clinical sample to determine viral circRNAs that are expressed *in vivo* and thus likely to be virologically relevant. Detection of elevated levels of KSHV circRNAs in human tissue samples would likely indicate lytically-infected cells, since many KSHV circRNAs are expressed exclusively in the lytic cycle. Amplicon-Seq of BSJs allows identification of highly utilized BSJs, hence dominant isoforms of viral circRNAs, which can be used for targeted knock-down in future functional studies. We showed that quantitating expression levels at the individual exon-level, rather than genes or transcripts, can reveal the effect on back-splicing and found that KSHV infection alters expression of certain human circRNAs in post-transcriptional manner. Furthermore, knocking-down a series of RNA-binding proteins revealed new RBPs that regulate expression of human and viral circRNAs. Combinations of novel techniques and analysis thus contributed to characterize interactions of herpesviruses and circRNAs.

Use of unconventional donor/acceptors and back-splicing were observed in a variety of infections. In the previous work, we cloned several amplicons of divergent primer RT-PCR and found that BSJs of several KSHV circRNAs are variable (Tagawa et al., 2018). Many viral circRNAs are located in genes that have a single exon and do not contain a previously known splice site (Tagawa et al., 2018; Toptan et al., 2018; Ungerleider et al., 2019, 2018; Zhao et al., 2019). Widely used splicing maps are possibly limited to detect these unconventional splice sites since they are often derived from short read sequencing like Illumina (Arias et al., 2014), high level of coverage, and the reliance on canonical human splice donor/acceptor sequences, which does not account for many back-splicing events. Use of long-read sequencing like Nanopore and appropriate mapping of BSJ-containing reads may help identify previously unseen transcript isoforms as seen in MHV68 mouse herpesvirus (O’Grady et al., 2019). To understand the variabilities in a more comprehensive manner in this work, we utilized deep-sequencing of these amplicons. We observed variabilities in KSHV and EBV circRNAs, but there are a limited number of dominant sites where BSJs are observed for specific circRNAs, such as circORF72 or circT0.7 (Figure 2). The mechanism by which these variable viral circRNAs are made is unknown. However, these current descriptions of dominant splice sites will allow targeted blocking of splicing using antisense oligo nucleotides (Yoshimoto et al., 2020) to find clues about function after repressing specific circRNAs during infections.

While the function of circvIRF4 is currently unknown, vIRF4 proteins have been reported to regulate p53 and Notch signaling (Baresova et al., 2013). However, circvIRF4 was identified to not interact with polysomes (Toptan et al., 2018), suggesting that peptides are unlikely generated from this circular RNA. Interestingly, the lymph node from the presented PEL patient with a poor clinical outcome had exceptionally high expression of circvIRF4. Further studies employing more clinical samples, optimally in longitudinal studies, may answer whether high expression levels of circvIRF4 routinely correlate with poor clinical outcomes.

We found BSJ-containing reads that were mapped in the KSHV latent transcript-encoding locus, which hosts multiple viral genes (protein-encoding and non-coding RNAs) including LANA, ORF72, and T0.7 (Ajiro and Zheng, 2014). Interestingly, T0.7 also encodes the microRNAs kshv-mir-K12-10 and mir-K12-12, which are conserved among KSHV-positive PEL cell lines and diverse patients from another study (Marshall et al., 2007). As the 5p and 3p of these miRNAs have complementary sequences^2^, miR-K12-10 and -12 have the potential to bind to circT0.7, which may trigger degradation by an RNA interference mechanism. Conversely, circT0.7 may trigger target-directed miRNA degradation (TDMD) in which targeted RNAs cause degradation of bound miRNAs. HCMV’s UL144-145, for example, was shown to degrade hsa-miR-17 and hsa-miR-20 (Lee et al., 2013). Human and viral circRNAs were found to sponge miRNAs to regulate their function (Huang et al., 2019; Kristensen et al., 2019; Liu et al., 2019) and thus it is possible that circT0.7 may regulate those viral miRNAs. It is unclear why it is beneficial for a virus to express then repress its own miRNAs, but it has a precedent: Simian virus 40 encodes miRNA to control its own T-antigen to evade T cell immunity (Sullivan et al., 2005). Gamma-herpesviruses establish infection for life, which necessitates efficient immune evasion, often with viral non-coding RNAs (Skalsky and Cullen, 2010; Tagawa et al., 2020). KSHV may have a similar system to fine-tune the viral miRNA expression or for viral miRNAs to target circT0.7 for degradation.

Human circRNAs are normally formed by back-splicing, but this does not happen in every intron-exon junction. Various RBPs have been reported to shift the balance between forward-splicing and back-splicing (Kristensen et al., 2019). We identified specific RBPs that can affect expression of such viral circRNAs, but the regulation of circRNA production depends on the specific interactions between some RBPs and circRNAs (Figure 5). We do not know where this specificity stems from, but sequence-specific binding of RBPs may be one of the reasons. These unanswered questions warrant further research to reveal the mechanism by which these viral circRNAs are produced.

In addition to RBP knock-down, we approached functional and mechanistic aspects of viral circRNAs with transcriptome analysis upon ectopic expression of viral circRNAs (Figure 6). The regulatory mechanism by which viral circRNAs regulate various mRNAs is curently unknown. We employed *in silico* predictions of miRNA binding to viral circRNAs as well as DEGs. We identified several miRNAs that can potentially be sequestered by viral circRNAs (Supplementary Table 7), warranting a future study. *PAX2* is a transcription factor that is induced by KSHV infection leading to better angiogenesis and invasion (Rivera-Soto and Damania, 2019). Both viral circRNAs we have tested reduced *PAX2* transcript levels significantly, suggesting their potential function in regulating uncontrolled invasion. As discussed for circT0.7 and viral miRNAs, fine-tuning function may be a common theme of viral circRNAs.

Currently no KSHV circRNAs are found to be associated with polysomes (Toptan et al., 2018; Abere et al., 2020b), but it is possible that these viral circRNAs regulate genes by interacting with other RNA species or RNA-binding proteins. Though a magnitude of transcriptomic change of single viral circRNA may be mild as seen in our volcano plots (Figure 6A), there can be significant change when all viral circRNAs are expressed simultaneously or in combination with their natural viral cofactors. The unconventional splicing of viral circRNAs is still a major obstacle for loss- and gain-of-function assays, but this work may serve as a foundation to investigate functions of viral circRNAs.

## Data Availability Statement

The datasets presented in this study can be found in online repositories. The names of the repository/repositories and accession number(s) can be found in the article/Supplementary Material.

## Ethics Statement

The studies involving human participants were reviewed and approved by Institutional Review Board approved protocol at the National Cancer Institute (NCTNCT00006518). The patients/participants provided their written informed consent to participate in this study.

## Author Contributions

TT, SD, and JZ designed the experiments. TT, DO, JS, and SD performed the experiments. TU and RY provided a clinical sample. VK and GM analyzed deep-sequencing data. TT and JZ interpreted data and wrote the manuscript. All authors contributed to the article and approved the submitted version.

## Conflict of Interest

The authors declare that the research was conducted in the absence of any commercial or financial relationships that could be construed as a potential conflict of interest.

## Footnotes

1 www.circbase.org

2 www.miRBase.org

## Supplementary Material

Supplementary Figure 1 Variable back-spliced junctions of KSHV and EBV circRNAs. Alignments of back-spliced junctions detected from KSHV and EBV positive cells. 5′ and 3′ ends are conjoined and set as 0. **(A)** An example of alignments of BSJ-containing reads or cloned cDNA. Sequences that can be chimerically mapped in head-to-tail manner on viral genomes are recognized as BSJ-containing reads. For panels **(B,C)**, viral chimeric back-spliced junction reads were collected from KSHV-infected HUVECs (Tagawa et al., 2018) and reactivated BCBL1 (Ungerleider et al., 2019) (left) and aligned to viral genomes (middle), then the furthest 5′ and 3′ ends were set to zero to conjoin the BSJ (right). **(B)** BSJ-containing reads at KSHV ORF34, 35-36, and 61 from KSHV-infected HUVECs, reactivated TREx-BCBL1 RTA (Tagawa et al., 2018), and reactivated BCBL1 (Ungerleider et al., 2019) were aligned at positions 54,776–55,156, 55,783–56,547, and 97,790–98,367 of NC_009333. Individual BSJ reads are shown as concentric circles with common sequences shared denoted as the dotted lines. Gaps in concentric circles are in fact joined in the BSJs reads, but the gaps depict the differences in sequences across viral circRNA variants. **(C)** 20 representative BSJ-containing reads from EBV positive cell lines (Ungerleider et al., 2019) are aligned at KSHV BHLF1 region (39,799–40,265 of NC_007605). All BSJ reads are listed in Supplementary Table 3.

Supplementary Table 1 Primers. List of primers utilized for RT-qPCR, ddPCR, and amplicon sequencing.

Supplementary Table 2 Alignment statistics. Statistics of detected back-splice junction- containing reads from RNA-Seq projects used here.

Supplementary Table 3 Detected circRNAs from RNA-Seq. Detected BSJ-containing reads by CIRCexplorer2 for RNA-Seq and circRNA-Seq. All detected BSJ reads from PEL5-LN, BCBL1 (GSM3543751), BC1 (GSM3309426), and triplicates of infected LEC (rK_LEC_siCon, PRJEB31253) as well as BSJ reads at EBV BHLF1 gene from Akata (GSM3258256 and GSM3258253), Jijoye (GSM3258264), JY (GSM3258265), Mutu I (GSM3258260 and GSM3258259), SNU719 (GSM3258268) cell lines are included.

Supplementary Table 4 BSJ detected from amplicons. Detected BSJ-containing reads by CIRCexplorer2 for amplicon-Seq.

Supplementary Table 5 SLK-circRNA DEG analysis. Differentially expressed gene analysis of SLK-circRNA cell lines by DESeq2.

Supplementary Table 6 circRNAs and RNA-binding proteins. Expression levels and contributions to circRNA production of genes that code for RNA-binding proteins [based on Queiroz et al. (2019) and Urdaneta et al. (2019)] are compiled based on publicly available data. Log2 Fold changes of gene expressions in KSHV-infected HUVECs (infected vs mock) are based on (Tagawa et al., 2018). Primary human umbilical vascular endothelial cells (HUVECs) were infected with KSHV. Effects on circRNA productions were determined by high-throughput screen and based on (Li et al., 2017).

Supplementary Table 7 Potential miRNAs sponged by viral circRNAs. TargetScan7.2 was utilized to predicted binding of human miRNAs to viral circRNAs as well as significantly upregulated gene upon ectopic expression of the viral circRNAs of interest (Figure 6 and Supplementary Table 5). Binding affinity of 7 mer or better was used as valid predictions. miRNA families that can bind both viral circRNAs and DEGs are listed.
