# Supplementary figures and images for: Characterizing Expression and Regulation of Gamma-Herpesviral Circular RNAs
Source: Front Microbiol. 2021 Jun 30;12:670542. doi: 10.3389/fmicb.2021.670542 (PMC8278476; doi:10.3389/fmicb.2021.670542)

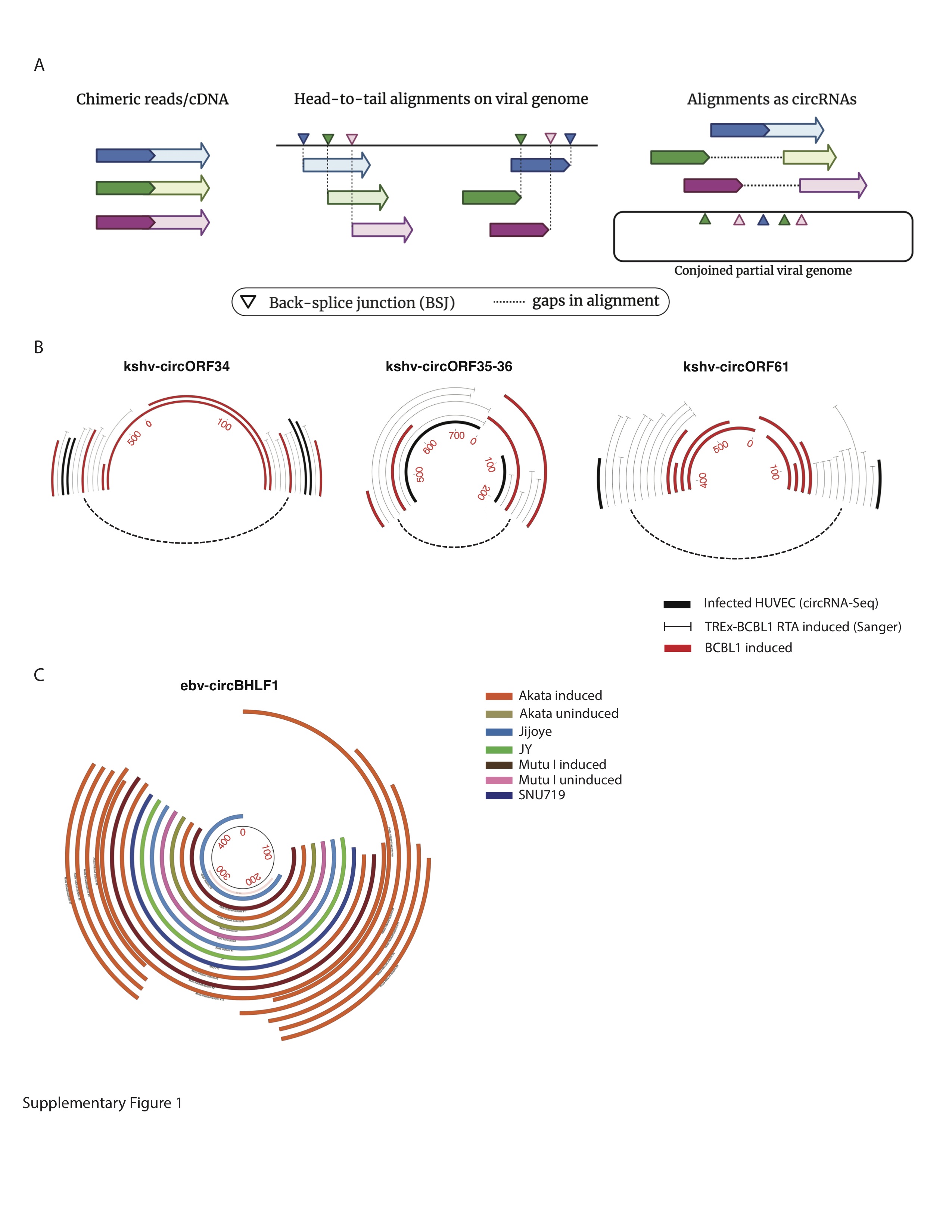

Supplement: Supplementary Figure 1 — Variable back-spliced junctions of KSHV and EBV circRNAs. Alignments of back-spliced junctions detected from KSHV and EBV positive cells. 5′ and 3′ ends are conjoined and set as 0. (A) An example of alignments of BSJ-containing reads or cloned cDNA. Sequences that can be chimerically mapped in head-to-tail manner on viral genomes are recognized as BSJ-containing reads. For panels (B,C), viral chimeric back-spliced junction reads were collected from KSHV-infected HUVECs (Tagawa et al., 2018) and reactivated BCBL1 (Ungerleider et al., 2019) (left) and aligned to viral genomes (middle), then the furthest 5′ and 3′ ends were set to zero to conjoin the BSJ (right). (B) BSJ-containing reads at KSHV ORF34, 35-36, and 61 from KSHV-infected HUVECs, reactivated TREx-BCBL1 RTA (Tagawa et al., 2018), and reactivated BCBL1 (Ungerleider et al., 2019) were aligned at positions 54,776–55,156, 55,783–56,547, and 97,790–98,367 of NC_009333. Individual BSJ reads are shown as concentric circles with common sequences shared denoted as the dotted lines. Gaps in concentric circles are in fact joined in the BSJs reads, but the gaps depict the differences in sequences across viral circRNA variants. (C) 20 representative BSJ-containing reads from EBV positive cell lines (Ungerleider et al., 2019) are aligned at KSHV BHLF1 region (39,799–40,265 of NC_007605). All BSJ reads are listed in Supplementary Table 3. [file Image_1.JPEG]
